# Supplementary material for: Human Sterols Are Overproduced, Stored and Excreted in Yeasts
Source: Int J Mol Sci. 2024 Jan 8;25(2):781. doi: 10.3390/ijms25020781 (PMC10815178; doi:10.3390/ijms25020781)
Supplement: Supplementary file 1 [file ijms-25-00781-s001.zip › ijms-2788101-supplementary.pdf]

Supplementary Figures and Tables

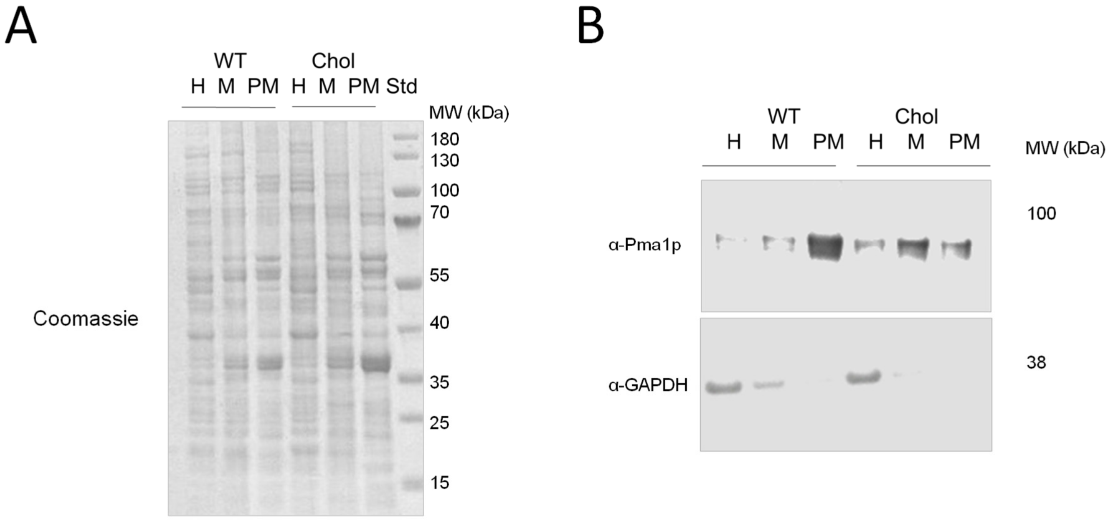

Figure S1: Immunoblot analysis of *K. phaffii* plasma membrane fractions. (A) The *K. phaffii* wild type and cholesterol strains were cultivated to middle exponential phase, harvested, disrupted and fractionated applying a density gradient centrifugation as described in Materials and Methods. Protein patterns of the homogenate (H), a mitochondria-enriched fraction (M) collected after the first density gradient centrifugation at the interphase 43% sucrose and TE-buffer, and purified plasma membrane fraction (PM) are shown. Molecular mass standards are indicated in the lane on the right (kDa). Lanes were loaded with 15 µg of total protein, each. Proteins were stained with Coomassie blue. (B) Quality control of the fractions was done by immunoblot analysis. Antisera were directed against plasma membrane H+-ATPase Pma1p, and cytosolic glyceraldehyde-3-phosphate-dehydrogenase GAPDH (Grillitsch et al., 2014).

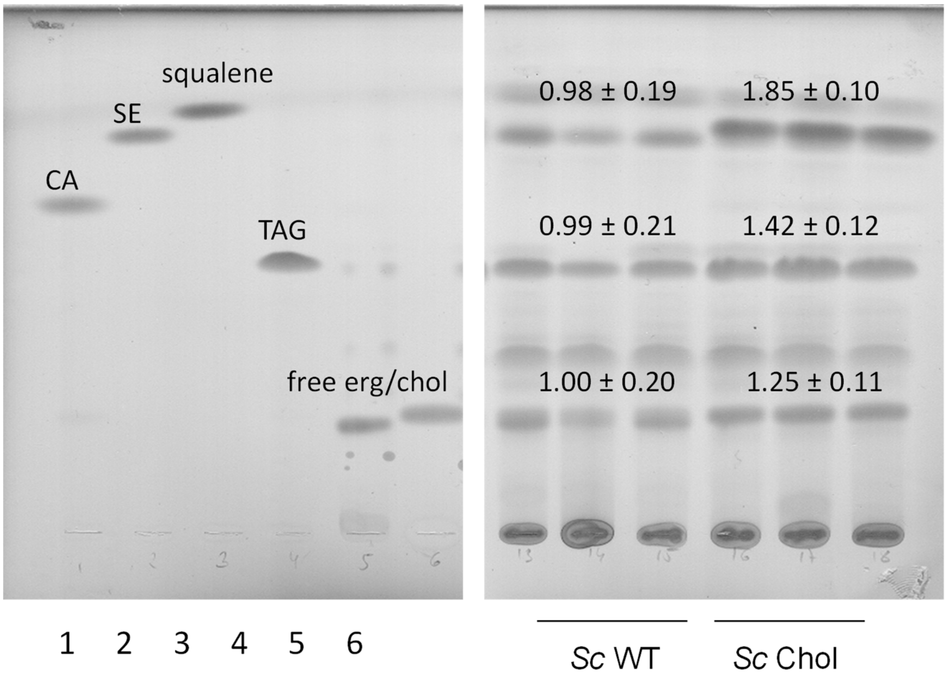

Figure S2: TLC analysis of wild type and cholesterol producing *S. cerevisiae* strains. The *S. cerevisiae* wild type and cholesterol strains were cultivated at 28°C for 24 h until they reached early exponential phase, harvested by centrifugation and analyzed by thin layer chromatography. Applied standards were 6 µg of (1) cholesteryl acetate (CA), (2) cholesteryl oleate/sterol ester (SE), (3) squalene, (4) triolein (TAG), (5) free ergosterol, and (6) free cholesterol. Band intensities obtained on TLCs were quantified using Fiji [1] and signal quantifications are presented as mean +/- standard deviations from three biological replicates.

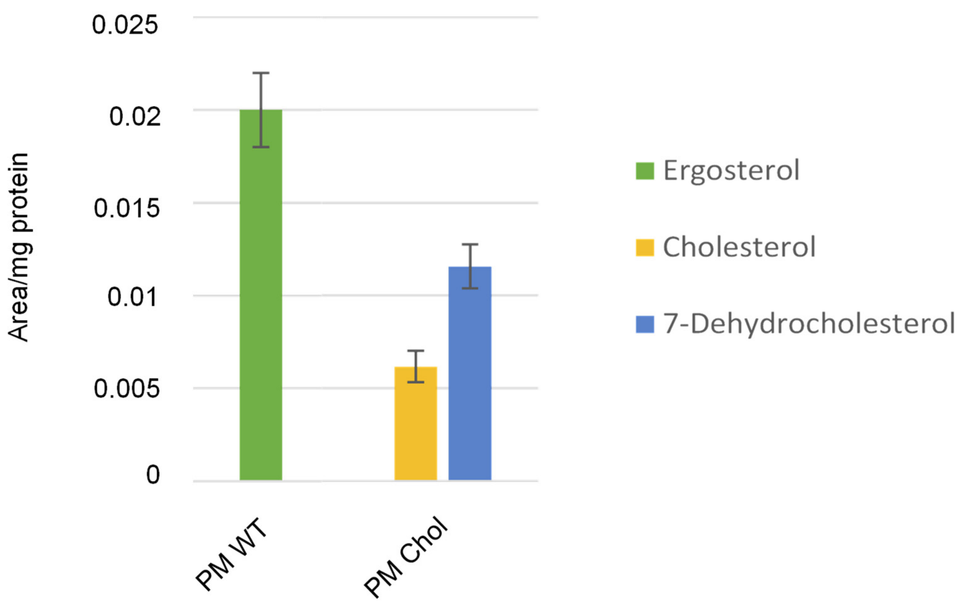

Figure S3: Sterol analysis of purified plasma membrane fractions of the wild type and cholesterol producing *K. phaffii* strain. *K. phaffii* wild type and cholesterol strains were cultivated at 28°C for 24 h until they reached early exponential phase and harvested by centrifugation. Plasma membrane (PM) fractions were purified, sterols were extracted and analyzed by GC-MS as described in Materials and Methods. Data are presented as mean from three biological replicates +/- standard deviations.

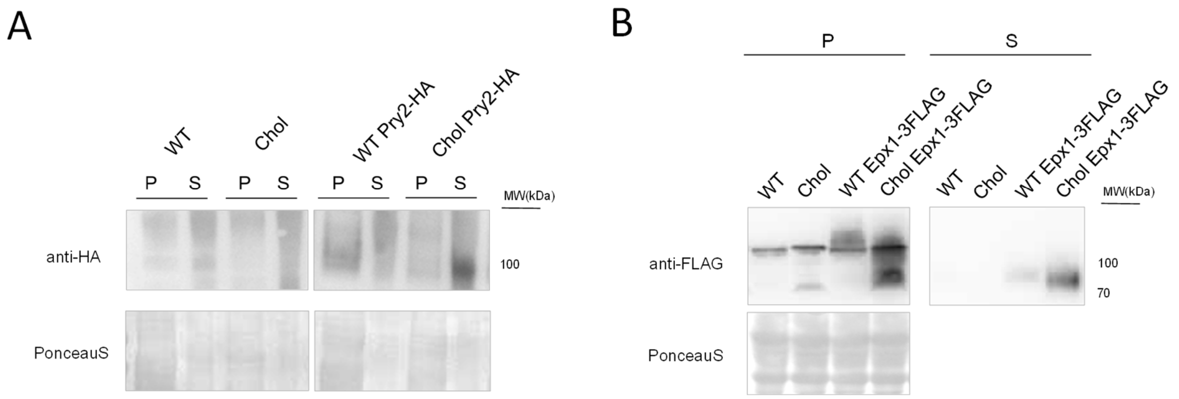

Figure S4: Pry2-HA and Epx1-3FLAG are overproduced and -secreted in the cholesterol strain. (A) A wild type *K. phaffii* strain, the cholesterol producing *K. phaffii* strain (yMH468) and a wild type producing Pry2-HA (yAR021) and a cholesterol strain producing Pry2-HA (yAR023) from its endogenous promoter were cultivated to middle exponential phase at 28°C, harvested, lysed, and proteins were extracted either from the cell pellets (P) or precipitated from the culture supernatant (S), resolved by SDS–PAGE, and analyzed by immunoblotting with anti-HA antiserum, as described under Materials and Methods. Loading control, PonceauS detected on the same immunoblots prior to blocking and incubation with antibody. MW, marker proteins (kDa). (B) A wild type *K. phaffii* strain, the cholesterol producing *K. phaffii* strain (yMH468) and a wild type producing Epx1-FLAG (yAR041) and a cholesterol strain producing Epx1-FLAG (yLB188) from its endogenous promoter were prepared for immunoblot analysis as described under (A) and analyzed with anti-FLAG® M2-Peroxidase (HRP) antibody. MW, marker proteins (kDa).

Table S1: Strategies and plasmids applied for strain generation

CRISPR/Cas9 strategies			
Purpose	sgRNA targeting site and PAM sequence (Cas9 plasmid)	Preparation of repair cassette	Yeast strain(s) generated
3HA-tagging of *PRY2*	ATCTACCAGTGGTAACACATTGG (pAR003, pPpHyg-Cas9-*PRY2-TT*)	Cut of pAR005 (pPpKC2-*PRY2*-3HA) with *Smi*I, purification of 2875 DNA band	yAR021, yAR023
3FLAG-His_6 tagging of *EPX1*	TTATCAGTGAGTCAGTCATCAGG (pAEA464, pPpHyg-Cas9-*EPX1-TT*)	*EPX1*-3FLAG-His_6 repair cassette generated by PCR	yAR041, yLB188
Frameshift mutation in *UPC2*	GCTCTGATCCAGGGATCTTTAGG (pAR015, pPpHyg-Cas9-*UPC2*)	-	yAR097
Frameshift mutation in *SNQ2*	CTCTCGCATAATAACAGCTCTGG (pFG011, pPpHyg-Cas9-*SNQ2*)	-	yAR070
Frameshift mutation in *PDR5*	GAATGGGTCTGATTCCGAATTGG (pAR007, pPpHyg-Cas9-*PDR5*)	-	yAR104, yAR105
Frameshift mutation in *PDR12*	TATGAGCCCACTAGATTTGTAGG (pFG013, pPpHyg-Cas9-*PDR12*)	-	yAR077
Frameshift mutation in *PDR15*	GCAGTGAAGAAGCCATTCAAAGG (pAR012, pPpHyg-Cas9-*PDR15*)	-	yAR093

Table S2: Yeast strains used in this study

Strain	Genotype	Reference or source
S. cerevisiae		
RH2881	*MAT***a** *ura3Δ leu2Δ his3Δ trp1Δ can1Δ bar1Δ*	Howard Riezman strain collection
RH6829	*MAT***a** *ura3Δ leu2Δ his3Δ trp1Δ can1Δ bar1Δ erg5*::*HIS3*-*TDH3*$_{prom}$-*DrDHCR24 erg6*::*TRP1*-*TDH3*$_{prom}$-*DrDHCR7*	[3]
K. phaffii		
CBS7435[a]	*his4Δ*	[4]
yMH468	CBS7435 *his4Δ erg5*::*GAP*$_{prom}$-*DrDHCR7*-Zeo^R *erg6*:: *GAP*$_{prom}$-*DrDHCR24*-G418^R	[5]
yAR021	CBS7435 *his4Δ PRY2*-3HA	This study
yAR023	CBS7435 *his4Δ erg5*::*GAP*$_{prom}$-*DrDHCR7*-Zeo^R *erg6*:: *GAP*$_{prom}$-*DrDHCR24*-G418^R *PRY2*-3HA	This study
yAR041	CBS7435 *his4Δ EPX1*- His$_6$-3FLAG	This study
yLB188	CBS7435 *his4Δ erg5*::*GAP*$_{prom}$-*DrDHCR7*-Zeo^R *erg6*:: *GAP*$_{prom}$-*DrDHCR24*-G418^R *EPX*- His$_6$-3FLAG	This study
yAR070	CBS7435 *his4Δ snq2-1*	This study
yAR056	CBS7435 *his4Δ pdr5-1*	This study
yAR077	CBS7435 *his4Δ pdr12-1*	This study
yAR093	CBS7435 *his4Δ pdr15-1*	This study
yAR097	CBS7435 *his4Δ erg5*::*GAP*$_{prom}$-*DrDHCR7*-Zeo^R *erg6*:: *GAP*$_{prom}$-*DrDHCR24*-G418^R *upc2-1*	This study
yAR074	CBS7435 *his4Δ erg5*::*GAP*$_{prom}$-*DrDHCR7*-Zeo^R *erg6*:: *GAP*$_{prom}$-*DrDHCR24*-G418^R *snq2-1*	This study
yAR053	CBS7435 *his4Δ erg5*::*GAP*$_{prom}$-*DrDHCR7*-Zeo^R *erg6*:: *GAP*$_{prom}$-*DrDHCR24*-G418^R *pdr5-1*	This study
yAR080	CBS7435 *his4Δ erg5*::*GAP*$_{prom}$-*DrDHCR7*-Zeo^R *erg6*:: *GAP*$_{prom}$-*DrDHCR24*-G418^R *pdr12-1*	This study
yAR092	CBS7435 *his4Δ erg5*::*GAP*$_{prom}$-*DrDHCR7*-Zeo^R *erg6*:: *GAP*$_{prom}$-*DrDHCR24*-G418^R *pdr15-1*	This study

[a] NRRL Y-11430, ATCC 76273.

Supplementary References

1. Schindelin, J.; Arganda-Carrera, I.; Frise, E.; Verena, K.; Mark, L.; Tobias, P.; Stephan, P.; Curtis, R.; Stephan, S.; Benjamin, S.; et al. Fiji - an Open Platform for Biological Image Analysis. *Nat. Methods* **2009**, *9*, doi:10.1038/nmeth.2019.Fiji.

2. Guan, X.L.; Riezman, I.; Wenk, M.R.; Riezman, H. Yeast Lipid Analysis and Quantification by Mass Spectrometry. *Methods Enzymol.* **2010**, *470*, 369–391, doi:10.1016/S0076-6879(10)70015-X.

3. Souza, C.M.; Schwabe, T.M.E.; Pichler, H.; Ploier, B.; Leitner, E.; Guan, X.L.; Wenk, M.R.; Riezman, I.; Riezman, H. A Stable Yeast Strain Efficiently Producing Cholesterol Instead of Ergosterol Is Functional for Tryptophan Uptake, but Not Weak Organic Acid Resistance. *Metab. Eng.* **2011**, *13*, 555–569, doi:10.1016/j.ymben.2011.06.006.

4. Näätsaari, L.; Mistlberger, B.; Ruth, C.; Hajek, T.; Hartner, F.S.; Glieder, A. Deletion of the *Pichia Pastoris KU70* Homologue Facilitates Platform Strain Generation for Gene Expression and Synthetic Biology. *PLoS One* **2012**, *7*, e39720, doi:10.1371/journal.pone.0039720.

5. Bernauer, L.; Berzak, P.; Lehmayer, L.; Messenlehner, J.; Oberdorfer, G.; Zellnig, G.; Wolinski, H.; Augustin, C.; Baeck, M.; Emmerstorfer-Augustin, A. Sterol Interactions Influence the Function of Wsc Sensors. *J. Lipid Res.* **2023**, *64*, 100466, doi:10.1016/j.jlr.2023.100466.
